# High-Resolution *En Face* Images of Microcystic Macular Edema in Patients with Autosomal Dominant Optic Atrophy

**DOI:** 10.1155/2013/676803

**Published:** 2013-11-28

**Authors:** Kiyoko Gocho, Sachiko Kikuchi, Takenori Kabuto, Shuhei Kameya, Kei Shinoda, Atsushi Mizota, Kunihiko Yamaki, Hiroshi Takahashi

**Affiliations:** ^1^Department of Ophthalmology, Nippon Medical School Chiba Hokusoh Hospital, 1715 Kamagari, Inzai, Chiba 270-1694, Japan; ^2^Department of Ophthalmology, Teikyo University School of Medicine, 2-11-1 Kaga, Itabashi-ku, Tokyo 173-8605, Japan; ^3^Department of Ophthalmology, Nippon Medical School, 1-1-5 Sendagi, Bunkyo-ku, Tokyo 113-8602, Japan

## Abstract

The purpose of this study was to investigate the characteristics of microcystic macular edema (MME) determined from the *en face* images obtained by an adaptive optics (AO) fundus camera in patients with autosomal dominant optic atrophy (ADOA) and to try to determine the mechanisms underlying the degeneration of the inner retinal cells and RNFL by using the advantage of AO. Six patients from 4 families with ADOA underwent detailed ophthalmic examinations including spectral domain optical coherence tomography (SD-OCT). Mutational screening of all coding and flanking intron sequences of the *OPA1* gene was performed by DNA sequencing. SD-OCT showed a severe reduction in the retinal nerve fiber layer (RNFL) thickness in all patients. A new splicing defect and two new frameshift mutations with premature termination of the Opa1 protein were identified in three families. A reported nonsense mutation was identified in one family. SD-OCT of one patient showed MME in the inner nuclear layer (INL) of the retina. AO images showed microcysts in the *en face* images of the INL. Our data indicate that AO is a useful method to identify MME in neurodegenerative diseases and may also help determine the mechanisms underlying the degeneration of the inner retinal cells and RNFL.

## 1. Introduction

Autosomal dominant optic atrophy (ADOA; MIM no. 165500), also known as Kjer's disease [1], is the most common hereditary ocular neuropathy with a prevalence of 1/12,000–1/50,000 [2–4]. ADOA is characterized by a decrease in the visual acuity that develops in childhood, temporal palor of the optic discs, centrocecal scotoma, and color vision defects [5, 6]. Histopathological studies of human eyes with ADOA showed diffuse atrophy of the retinal ganglion cell (RGC) layer that predominated in the central retina [7, 8].

ADOA has considerable intra- and interfamilial clinical variability with incomplete penetrance estimated to be about 90% in the familial forms of the disease [9]. Mutations in the optic atrophy 1 gene, *OPA1* (MIM no. 605290), located on chromosome 3q28-q29, are responsible for about 60–80% of the cases of ADOA [10–12].

*OPA1* encodes a mitochondrial dynamin-related GTPase, which is anchored to the mitochondrial inner membrane [13, 14]. Although the Opa1 protein is ubiquitously expressed in human tissues, a strong expression of the Opa1 protein has been reported in the RGC layer [15]. The Opa1 protein has multiple functions and plays a key role in the fusion of mitochondria and thus in organizing the mitochondrial network [13, 14]. The other functions of the Opa1 protein are related to oxidative phosphorylation, maintenance of the membrane potential [11, 16, 17], maintenance of mtDNA [18, 19], organizing the cristae, and control of mitochondrial apoptosis through the compartmentalization of cytochrome C [17, 20]. Mutations of the *OPA1* gene result in a loss of function in most ADOA patients indicating that haploinsufficiency is involved in the pathomechanism of the disease [21]. However, to date, there is no clear evidence to suggest a further role for the *OPA1* gene in the degeneration of RGCs in ADOA.

Recently, Barboni et al. detected “microcystic macular edema (MME)” in the inner nuclear layer (INL) of patients with Leber's hereditary optic neuropathy (LHON) and ADOA [22]. The INL is predominantly made up of the nuclei of the horizontal, bipolar, and amacrine cells. MME was originally identified in patients with multiple sclerosis (MS) by Gelfand et al., and it was characterized by cystic lacunar areas of hyporeflectivity with clear boundaries in the spectral domain optical coherence tomographic (SD-OCT) images [23]. They suggested that MME represented a breakdown of the blood-retina barrier caused by subclinical uveitis or retinitis. Abegg et al. noted similar changes in a case of compressive optic neuropathy due to a glioma, but they suggested retrograde transsynaptic degeneration as the cause of MME [24]. Balk et al. noted similar characteristics in a case of recurrent optic neuritis not due to multiple sclerosis adding inflammation as a possible cause of MME [25].

Adaptive optics (AO) technology has enabled clinicians to view the retina with high microscopic lateral resolution [26, 27]. This technique has been used to analyze the cone photoreceptor mosaic in eyes with inherited retinal degenerations [28, 29]. It has also been used to analyze the inner retinal layers, for example, the retinal nerve fiber layer [30]. However, this new technology has not been used to analyze the inner layers of the retina in patients with MME. AO has a transverse resolution of approximately 1.6 *μ*m compared to commercial OCT systems with a resolution of approximately 15 *μ*m. This higher resolution should help in detecting and evaluating *en face* images of MME.

Thus, the purpose of this study was to investigate the characteristics of MME determined from the *en face* images obtained by an AO fundus camera in patients with ADOA and also to try to determine the mechanisms underlying the degeneration of the inner retinal cells and RNFL by AO. To accomplish this, 6 patients from 4 families with the *OPA1* gene were studied.

## 2. Methods

The protocol of this study conformed to the tenets of the Declaration of Helsinki and was approved by the Institutional Review Board of the Nippon Medical School. Six consecutive cases of ADOA patients from 4 families who visited Nippon Medical School Chiba Hokusoh Hospital from December 2010 through April 2013 were studied. A written informed consent was obtained from the six patients after an explanation of the nature and possible complications of the experimental protocol.

### 2.1. Clinical Examinations

The ophthalmological examinations included measurements of the best-corrected visual acuity (BCVA), determination of the refractive error (spherical equivalent), slit-lamp biomicroscopy, ophthalmoscopy, fundus photography, fluorescein angiography (FA), perimetry, SD-OCT, infrared imaging, and full-field electroretinography (ERG). The visual fields were obtained by Goldman perimetry and Humphrey Visual Field Analyzer (Model 745i; Carl Zeiss Meditec, Inc., Dublin, California). The Swedish interactive threshold algorithm standard strategy was used with program 30-2 of the Humphrey Visual Field Analyzer. Color vision was evaluated with the Farnsworth Panel D-15. SD-OCT (Carl Zeiss Meditec) images were obtained from all of the patients. The B-scan retinal images were composed of 27,000/s consecutive A-scans acquired through the center of the macula horizontally for Figures 5(b) and 8(a). In all patients, the fixation was centered on the macula. For Figure 8(b), we moved a horizontal scan line manually to the area containing the MME detected by AO with centered patient fixation. For RNFL thickness analysis, we performed a vertical SD-OCT scan at about 1 mm from the edge of optic disc with centered fixation. The total scan depth was 2 mm, the axial resolution was 5 *μ*m, and transverse resolution was 15 *μ*m. The images presented are 6-mm-long scans except for Figure 5(b) which has been cut to fit AO images. The 512 × 128 Macular Cube scan protocol was used to obtain the *en face* OCT images. With this protocol, 128 cross-sectional B-scan images were obtained, each composed of 512 A-scans. In all patients, fixation was centered on the macula. Full-field scotopic and photopic ERGs were recorded using an extended testing protocol incorporating the International Society for Clinical Electrophysiology of Vision standards [31].

### 2.2. Genetic Testing

Blood samples were collected from the patients, and genomic DNA was isolated from peripheral white blood cells with a blood DNA isolation kit (NucleoSpin Blood XL; Macherey Nagel, Germany). The DNA was used as a template to amplify the *OPA1* gene. Coding regions and flanking introns of the *OPA1* gene were amplified by polymerase chain reaction (PCR) with published primers [32]. The PCR products were purified (ExoSAP-IT; USB Corp., USA), and both strands of the gene were sequenced with an automated sequencer (Bio Matrix Research; Chiba, Japan).

RT-PCR was used to amplify the cDNAs of *OPA1*. The mRNAs were obtained from peripheral white blood cells with the TRIzol reagent (Invitrogen, CA, USA), and template cDNAs were generated with random hexamer primers. We designed exon-spanning primer pairs and used them to amplify exon 18 to exon 20 of the *OPA1* cDNA. They are forward primer (5′-GTTGAACAACAGGCTGATAG-3′) and reverse primer (5′-GCTTGATATCCACTGTGGTG-3′). The recovered DNAs were subcloned into the StrataClone PCR cloning vector (Stratagene; CA, USA). Plasmid DNAs from 20 positive clones were purified with the Qiagen Plasmid Purification Kit (Qiagen, CA, USA) and sequenced with an automated sequencer (Bio Matrix Research; Chiba, Japan).

### 2.3. Adaptive Optics (AO) Flood Illumination Image Acquisition

Fundus images were obtained with an infrared AO retinal camera (rtx1, Imagine Eyes, Orsay, France) [33]. This system was used in earlier investigations to image individual cone photoreceptors [27, 29, 34, 35] and other retinal structures [27, 36]. In our study, the AO instrument illuminated a 4-degree square field of the retina with 850 nm infrared flashes to acquire *en face* images of the retina with a transverse optical resolution of 250 line pairs/mm. Successive AO images were taken at adjacent retinal locations with an angular spacing of 2 degrees in the horizontal and vertical directions. This procedure allowed for a horizontal and vertical overlap of at least 2 degree between successive images. Prior to each acquisition, the focusing depth was adjusted to the inner nuclear layer. The resulting images were stitched together by superimposing retinal vessel landmarks with an image editing software (GIMP, The GIMP Development Team; Image J, National Institute of Health, Bethesda, MD). The size of each pixel was typically 0.8 *μ*m when calculated at the retinal plane, and the values were adjusted for variations in the axial length of the eye [37]. We also analyzed normal controls and patients with advanced glaucoma to determine whether MME was present. They were 50 normal controls and 5 advanced glaucomatous retinas. There were 27 men and 23 women whose age ranged from 18 to 57 years (mean, 38.1 ± 8.3 years) in this normal control group. There were 3 men and 2 women whose age ranged from 37 to 57 years (mean, 46.8 ± 6.5 years) in the glaucoma group. The focusing depth was adjusted to the INL.

## 3. Results

### 3.1. Clinical Findings

**Figure 1:**
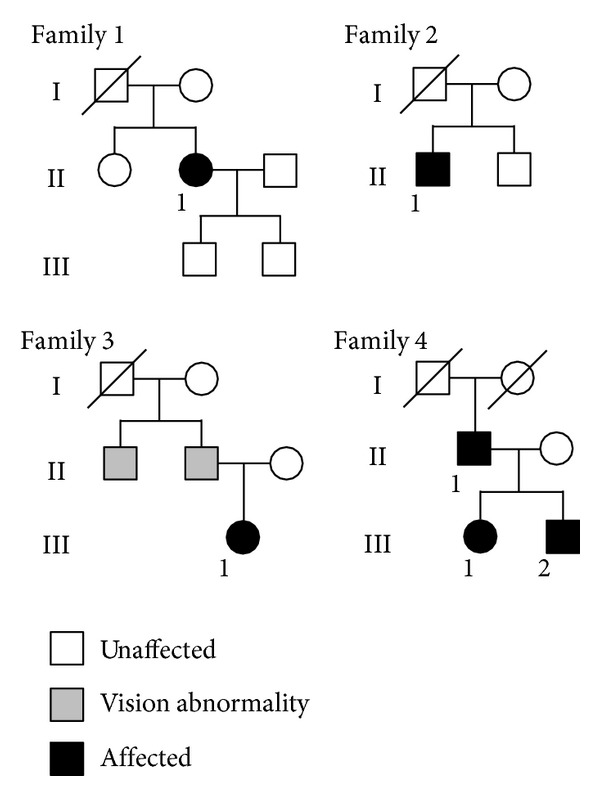
Pedigrees of the four families of six ADOA patients. Affected patients are shown with solid symbols and unaffected with open symbols. In family 3, two members who may have had vision abnormalities are shown with gray symbols. We were not able to examine them.

We studied 6 patients from 4 families with ADOA, (Figure 1) and the clinical characteristics of these 6 patients are summarized in Table 1. The decimal BCVA of all patients was reduced with a range from 0.7 to 0.07. The Goldmann kinetic visual fields showed a centrocecal scotoma in three patients and a blind spot enlargement in the other three patients. Temporal optic disc palor was seen in all patients. Ito et al. reported that the retinal nerve fiber layer (RNFL) in the macular area of patients with ADOA was significantly thinner than that in control subjects by SD-OCT [38]. They also showed that the RNFL in the temporal areas of circular scans around the optic disc was almost lost while the nasal areas were relatively well preserved.

**Figure 2:**
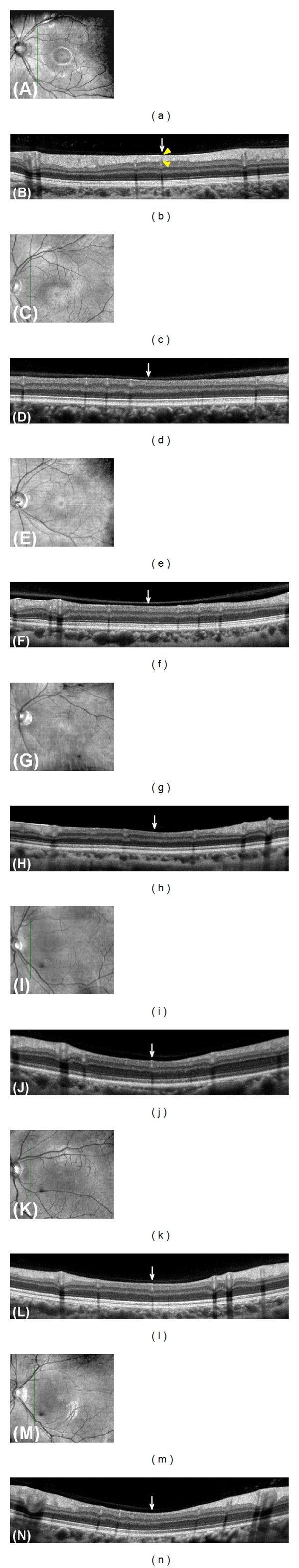
Retinal nerve fiber layer thickness analysis on spectral-domain optical coherence tomography (SD-OCT) images of the eyes in a normal control and in the ADOA patients. Infrared (IR) reflectance images (a, c, e, g, i, k, m) and SD-OCT images (b, d, f, h, j, l, n) are shown. The green vertical lines in the IR images indicate localization of scanned line to obtain the SD-OCT images. SD-OCT scan was performed from lower to upper retina. Images obtained from normal control (a, b), Patient 1-II-1 (c, d), Patient 2-II-1 (e, f), Patient 3-III-1 (g, h), Patient 4-II-1 (i, j), Patient 4-III-1 (k, l), and Patient 4-III-2 (m, n) are shown. Arrows indicate the temporal region of their optic disc. Note that the RNFL thickness (yellow arrowheads) of normal control is thick enough to measure in the temporal region of optic disc, while that of all ADOA patients is almost absent and appears as a thin line.

We performed a vertical SD-OCT scan at about 1 mm from the edge of optic disc. The results in the ADOA patients showed that the temporal RNFL was very thin in all of the patients (Figure 2). FA did not show any leakage in Patients 1-II-1 and 2-II-1 (data not shown). We did not perform FA on the other 4 patients.

**Figure 3:**
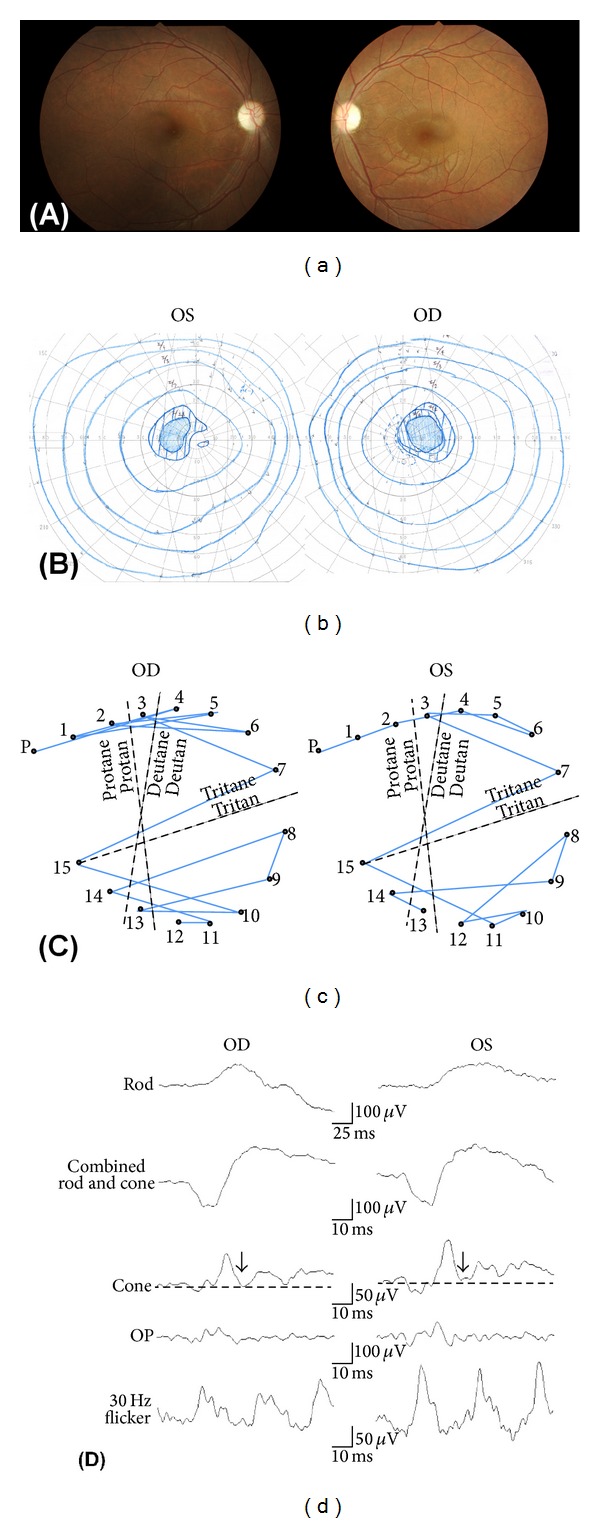
The clinical findings of Patient 1-II-1. (a) Fundus photograph of the patient showing temporal palor of the optic discs. (b) Goldmann kinetic visual fields showing bilateral centrocecal scotoma. (c) Panel D-15 shows that the confusion pattern is consistent with tritan (blue-yellow defect) axis in each eye. (d) Rod, combined rod-cone, cone, oscillatory potentials, and 30-Hz flicker full-field electroretinograms (ERGs) are shown. Photopic negative response (PhNR) of cone ERGs is reduced, and the peak of the PhNR is a positive potential relative to the baseline (dotted line). Arrows indicate PhNR.

The clinical findings of a representative case are shown in Figure 3 (Patient 1-II-1). Fundus examinations showed temporal palor of the optic discs (Figure 3(a)). A centrocecal scotoma was observed in the Goldmann kinetic visual fields test (Figure 3(b)). Panel D-15 showed that the confusion pattern was consistent with a tritan axis, blue-yellow defect in each eye (Figure 3(c)). The a- and b-waves of the scotopic and photopic full-field ERGs were of normal amplitudes. The amplitudes of the photopic negative response (PhNR) of the cone ERGs which is believed to originate from inner retinal layers have been reported to be reduced in ADOA patients [39]. In this case, the PhNR of the cone ERG was decreased, and the peak of the PhNR was a positive potential relative to the baseline (Figure 3(d)).

### 3.2. Molecular Genetic Findings

**Figure 4:**
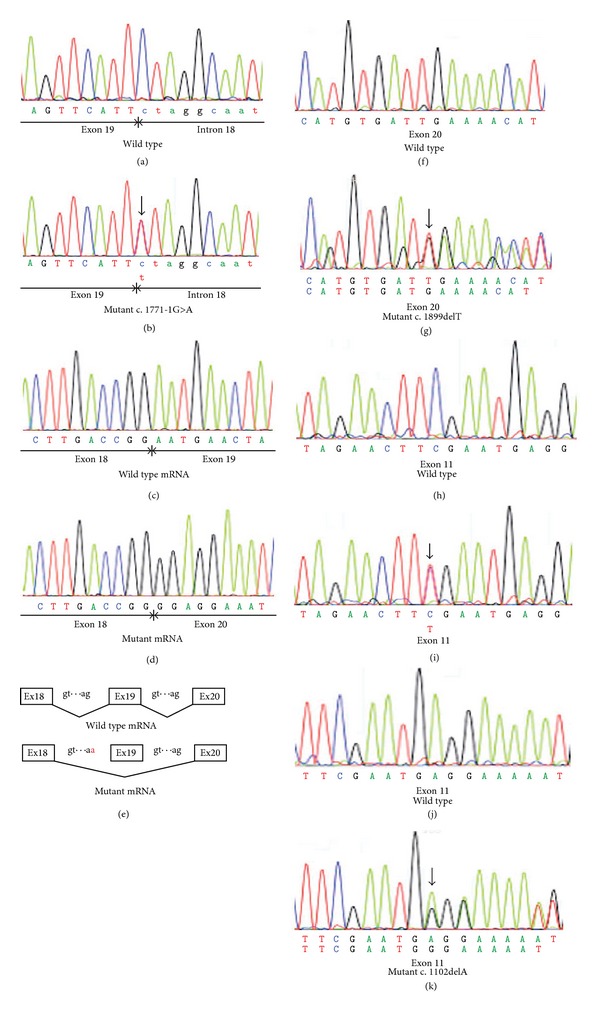
Molecular genetic findings of the ADOA patients. ((a) and (b)) Sequence chromatograms of the wild-type allele and the mutant allele (Patient 1-II-1) are shown. In the mutant allele (b), a heterozygous C to T (reverse strand) mutation, indicated by a vertical arrow, is shown at the −1 position of intron 18 (c.1771-1G>A). ((c) and (d)) Sequence chromatograms of the wild-type and the mutant (Patient 1-II-1) cDNAs from white blood cells are shown. Entire exon 19 is skipped in the mutant mRNA (d). Skipping exon 19 leads to a deletion of 77 bp of mRNA of *OPA1* gene and a resulting frameshift in the product (p.N591GfsX18). (e) Schematic diagram of the splicing error in Patient 1-II-1 is shown. As a result of G to A mutation at position −1 of intron 18, whole exon 19 is skipped in the mutant gene. ((f) and (g)) Sequence chromatograms of the wild-type allele and the mutant allele (Patient 2-II-1) are shown. In the mutant allele (g), a heterozygous one base-pair deletion indicated by a vertical arrow can be seen (c.1899delT). ((h) and (i)) Sequence chromatograms of the wild-type allele and the mutant allele (Patient 3-III-1) are shown. In the mutant allele (i), a heterozygous C to T mutation, indicated by a vertical arrow, is shown (c.1096C>T). ((j) and (k)) Sequence chromatograms of the wild-type allele and the mutant allele (Patient 4-II-1) are shown. In the mutant allele (g), a heterozygous one base-pair deletion, indicated by a vertical arrow, is shown (c.1102delA).

We identified one already reported pathogenic mutation and three new mutations in the four families (Table 2). Patient 1-II-1 was found to have a new heterozygous G to A mutation at position −1 of intron 18 that is likely to abolish the 3′ splice acceptor site (c.1771-1G>A; Figures 4(a) and 4(b)). The family history revealed no other members including her parents with any eye disease. We could not test the genetics in other family members because she was not willing to have them tested. Although this mutation has never been reported, a mutation at position −2 of intron 18 (c.1771-2A>G) has been reported to be pathogenic with a splicing defect [40].

To investigate the impact of the splice acceptor site mutation, we analyzed *OPA1 *transcripts expressed in the white blood cells from this patient. Two distinct RT-PCR products were obtained from the patient (data not shown). To separate the mutant transcripts from wild-type transcripts, the RT-PCR products were cloned into a cloning vector. Twenty clones from the patient were sequenced to verify the inserts, and 5 of them showed truncated inserts with a skipping of exon 19 (Figures 4(c)–4(e)). This skipping would yield a truncated protein with a premature termination codon due to a frameshift (p.N591GfsX18).

Patient 2-II-1 was found to have a new heterozygous single base-pair deletion at position 1899 (c.1899delT). This would yield a truncated protein with a premature termination codon due to a frameshift (p.I633MfsX12). We could not test the genetic changes in other family members because they were not willing to have them tested. Although this mutation is a novel mutation, two small deletion mutations within the same exon (c.1881_1882delAG, c.1892_1893delAT) have been reported as pathogenic mutations for ADOA [40, 41].

Patient 3-III-1 was found to have a reported nonsense mutation. A heterozygous C to T mutation at position 1096 (c.1096C>T) directly changed an arginine at amino acid position 366 to a stop codon (R366X). This mutation was confirmed to be pathogenic by several studies [10, 42, 43].

Patients 4-II-1, 4-III-1, and 4-III-2 from the same family were found to have a new heterozygous single base-pair deletion at amino acid position 1102 (c.1102delT) that would yield a truncated protein with a premature termination codon due to a frameshift (p.R368GfsX4).

All three new mutations identified caused a frameshift with premature termination codon. These mutations would likely be pathogenic by the mechanism of haploinsufficiency as reported [21].

### 3.3. High-Resolution Imaging of Microcystic Macular Edema (MME) by SD-OCT and Adaptive Optics

**Figure 5:**
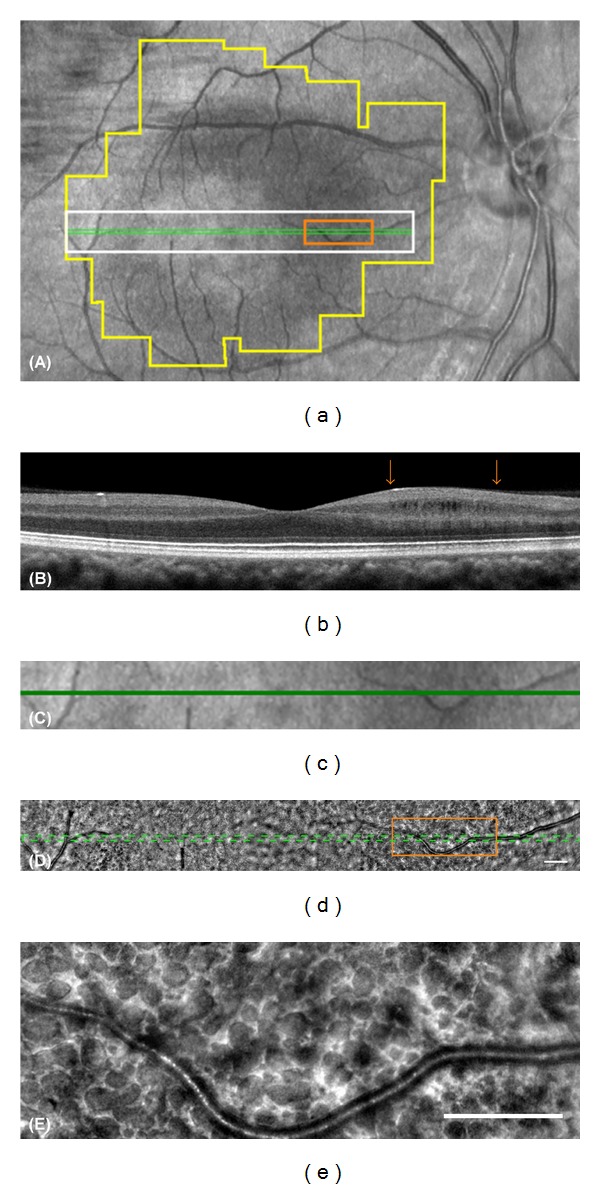
The localization and the structure of microcystic macular edema in Patient 1-II-1. (a) An infrared image of the macular region of the patient. The box outlined in green lines shows the area scanned to obtain the OCT image in (b). A white box indicates the area shown in (c) and (d). An orange box indicates the area shown in (e). A polygonal area outlined in yellow is the area shown in Figure 6(a). (b) SD-OCT image of the patient shows cystic lacunar areas of hyporeflectivity with clear boundaries in the nasal region. The RNFL is almost lost in this area. Arrows indicate the edge of the area outlined in orange in (a) and (d). (c) Magnified infrared image outlined in white in (a) is shown. A green line indicates the area scanned to obtain OCT image (b). (d) Montage of AO image corresponding to area (c) is shown. Note that retinal blood vessels are shown in exactly the same region in the images (c) and (d). (e) Magnified AO image outlined in orange in (a) and (d) is shown. The AO image shows various size dark reflectance areas outlined by hyperreflective region and most are oval shaped. Bars in (d) and (e) indicate 200 *μ*m.

**Figure 6:**
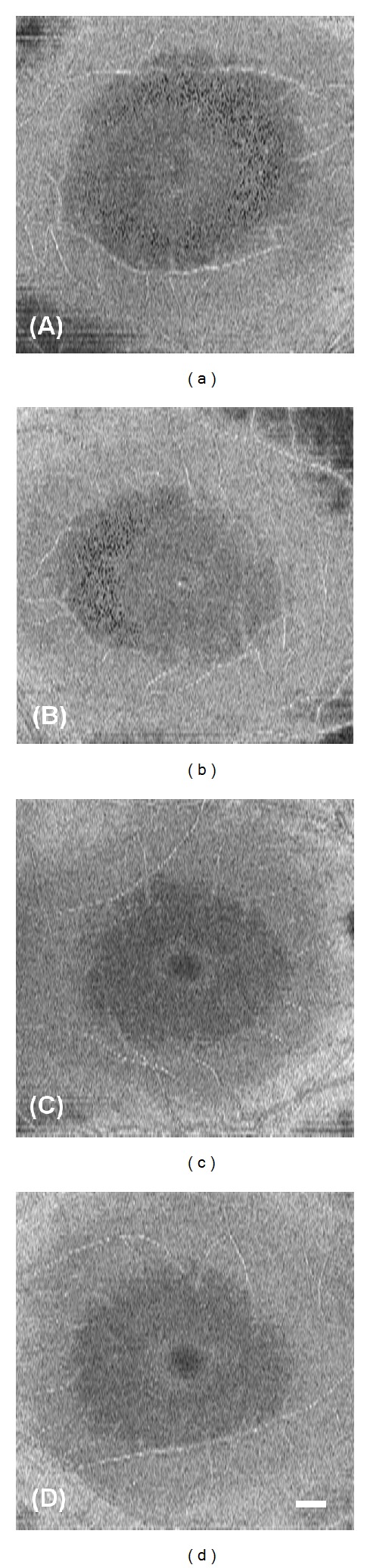
*En face* OCT images of Patient 1-II-1 and 2-II-1. *En face* SD-OCT images of the eyes in patient 1-II-1 ((a) and (b)) and 2-II-1((c) and (d)) are shown. Images from right eyes ((a) and (c)) and left eyes ((b) and (d)) are shown. *En face* OCT imaging reveals the presence of the cysts in patient 1-II-1. *En face* OCT images of the patient 2-II-1 did not show cysts clearly. Bars in (d) indicates 500 *μ*m.

The SD-OCT images of Patient 1-II-1 showed cystic lacunar areas of hyporeflectivity with clear boundaries, or MME, which were compatible with the characteristics suggested by Gelfand et al. (Figure 5(b)) [23]. Wolff et al. [44] reported that microcysts could also be observed using *en face* OCT imaging. *En face* OCT imaging of Patient 1-II-1 revealed that cysts were located in the superior, nasal, and inferior macular quadrants in both eyes (Figures 6(a) and 6(b)). AO imaging obtained from the same area showed high-resolution *en face* images of the microcysts in the inner layer of the retina (Figures 5(d) and 5(e)). They were of various sizes and appeared as dark reflectance areas outlined by hyperreflective regions. Most were oval shaped (Figures 5(d) and 5(e)). Similar structures were not found in more than 50 normal controls and 5 patients with advanced glaucoma in our department. We have not examined a patient with MS or optic nerve atrophy other than those with ADOA.

**Figure 7:**
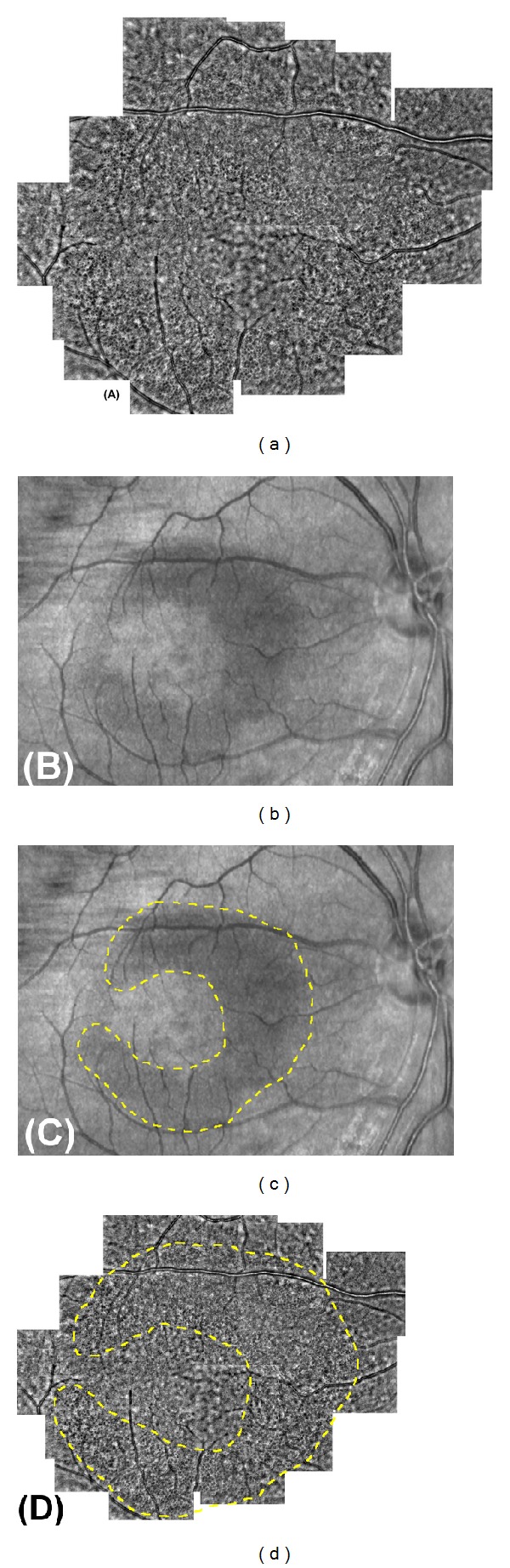
The AO and IR images of Patient 1-II-1. (a) Montage of AO images of the patient is shown. The microcystic structures are observed as perimacular rings. ((b) and (c)) Infrared (IR) images of the case show a hyporeflective region with perimacular ring shape. Perimacular ring shape is outlined by dotted yellow line (c). (d) Minimized image of that shown in (a). The area with microcystic structures is outlined in dotted yellow line. Note that the hyporeflective region in the IR image and the area containing microcystic structure in AO image are well matched.

Abegg et al. and Wolff et al. have reported that an area with MME is seen with different patterns as hyporeflective regions in the IR images in the perimacular area [24, 44]. The IR images of our ADOA cases also had ring-shaped hyporeflective regions in the perimacular area (Figures 5(a), 7(b), and 7(c)). The hyporeflective region in the IR image coincided well with the area containing the microcystic structures in the AO images (Figures 7(a)–7(d)).

**Figure 8:**
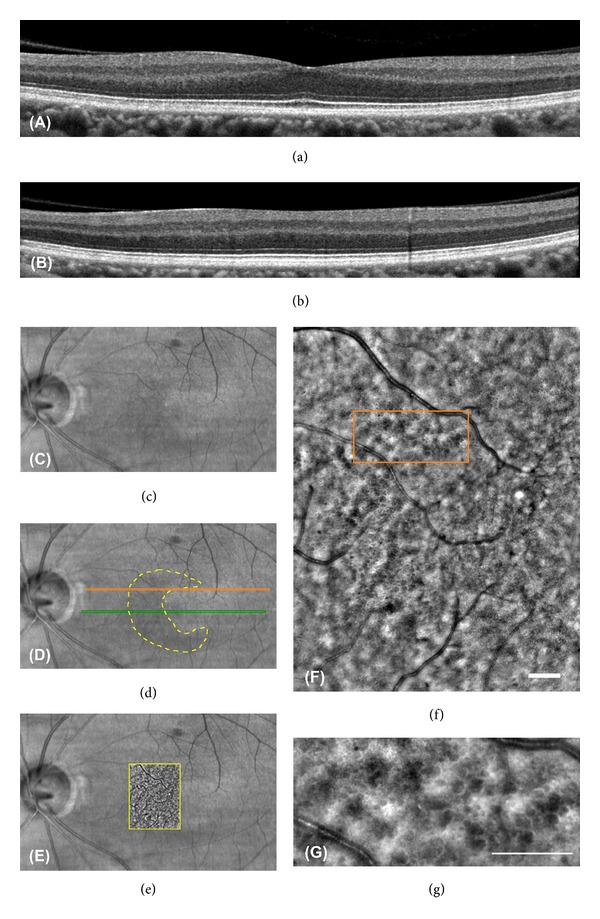
The OCT, IR, and AO images of Patient 2-II-1. ((a) and (b)) SD-OCT images of the patient do not show microcystic macular edema clearly. The RNFL is very thin in this area. The scan lines to obtain these images are shown in (d). ((c) and (d)) IR images of the patient are shown. The IR image has a crescent shaped perimacular hyporeflectance region outlined in yellow dotted line. The green and orange lines indicate the scan lines to obtain SD-OCT images of (a) and (b), respectively. The orange scan line overlaps the region outlined in (f). (e) IR image superimposed on AO image is shown. (f) Montage of AO images of the patient outlined area in (d) is shown. A small number of the microcystic structures are observed in the image. (g) Magnified AO image outlined in orange in (e) is shown. The AO image has various size dark reflectance areas outlined by hyperreflective region as observed in Patient 1-II-1. Bars in (e) and (f) indicate 200 *μ*m.

B-scan and *en face* SD-OCT images of Patient 2-II-1 did not show the MME clearly; however, the IR image had a crescent-shaped perimacular hyporeflective region (Figures 6(c), 6(d), and 8(a)–8(d)). In the crescent-shaped area, the *en face* AO image showed microcystic structures (Figures 8(e)–8(g)). The number of microcysts was fewer in Patient 2-II-1 than in Patient 1-II-1; however, the clarity of the microcysts was the same in these two patients (Figures 5(e) and 8(g)).

Although we analyzed the other 4 patients extensively, we did not find microcystic structures in their AO images. The refractive error and axial length were not significantly different in all six patients.

## 4. Discussion

Over 200 mutations in the *OPA1* gene have been identified in patients with ADOA (HGMD professional, Institute of Medical Genetics in Cardiff). Approximately one-half of the *OPA1* mutations lead to premature termination codons from nonsense mutations or frameshifts from small insertions, deletions, or splice site mutations [45]. These truncated mRNAs are unstable and get degraded by specific pathways, that is, nonsense-mediated mRNA decay, which are in-built protective cellular mechanisms against mutant proteins with possible dominant negative effect [42, 46, 47]. The reduced Opa1 protein expression levels observed in these reported cases support the role of haploinsufficiency in ADOA. These results strongly suggest that the three new heterozygous mutations with premature termination codon identified in this study are pathogenic.

Gelfand et al. reported that MME was associated with lower visual acuity and a thinner RNFL in patients with MS [23]. In our cases, Patient 1-II-1 with the poorest BCVA had the clearest MME in her SD-OCT and AO images. Our vertical SD-OCT image between the optic disc and macular region showed that the temporal RNFL was almost completely absent in all of the patients. However, among these patients, Patient 1-II-1 had the thinnest RNFL in the peripheral region of the vertical scan. Our data are consistent with the hypothesis that the degree of MME is related to the disease severity.

Gelfand et al. hypothesized that the presence of MME was associated with a breakdown of the blood-retinal barrier [23]. However, Barboni et al. noted that patients with LHON and DOA do not have any fluorescein leakage as expected for the noninflammatory status of their disease [22]. Our results also showed that genetically identified ADOA patients with MME do not have any signs of leakage from their retinal vessels.

MME has been detected in the INL of the retina with chiasmal glioma [24]. It is highly unlikely that the MME in a patient with brain tumor is due to inflammation of the retina and optic nerve. Thus, Abegg et al. hypothesized that the MME in the INL was due to retrograde transsynaptic degeneration [24].

It is well established that retrograde transsynaptic degeneration can occur in the human central nervous system [48, 49]. Van Buren observed atrophy of the RGC following a right occipital lobectomy in monkeys [50]. Recently, Jindahra et al. presented evidence of retrograde trans-synaptic degeneration of RGCs identified by SD-OCT following both congenital and acquired lesions of the retrogeniculate visual pathway in humans [51]. In addition, Green et al. reported that the neurodegenerative changes caused by retrograde transsynaptic degeneration in a patient with MS were seen not only in the RNFL and ganglion cell layer but also in the INL of their retina [52]. Their histopathological study showed prominent atrophy of the INL in 40% of the eyes suffering from MS and none of the control eyes. They also recognized that the severity of the INL atrophy appeared to be related to the severity of RGC atrophy. Similar INL vacuoles have been observed histopathologically in rhesus monkeys with idiopathic optic atrophy [53]. Combining these observations with our observations, we suggest the possibility that the dark regions observed in the *en face* AO images of our ADOA patients are areas of degenerated horizontal, bipolar, and amacrine cells in the INL caused by retrograde transsynaptic degeneration.

The *en face* MME structures detected by AO were also found in another ADOA patient who did not show MME clearly in the *en face* and cross-sectional OCT images. These observations indicate that AO might be useful in identifying MME in other neurodegenerative diseases and may also be helpful in determining the mechanisms underlying RGC and INL degeneration.

Our study has a number of limitations. We identified the *en face* MME in patients with ADOA; however, there are several other diseases that have MME in their SD-OCT images, for example, MS, recurrent optic neuritis, neuromyelitis optica, LHON, and chiasmal glioma. We need to investigate the *en face* MME structures in patients with such diseases to identify whether they also show the *en face* MME in their INL and to compare their features to those of *en face* MME seen in our patient with ADOA. It will probably be helpful in clarifying the pathomechanisms of the degeneration of inner retinal cell degeneration to investigate several diseases with different etiology.

We have found MME in the INL of the ADOA patients, but it is important to note that only in two patients. The cross-sectional nature of our study did not allow us to draw conclusions regarding the evolution of MME in ADOA and the other diseases. To address these issues, systematic longitudinal studies incorporating detailed ophthalmologic assessments in large cohort are needed and may help determine the mechanisms involved in the development of MME. Although the controls in our study including those with advanced glaucoma did not show MME in their *en face* AO images, we need to determine why patients with advanced glaucoma did not show *en face *MME despite the RGC loss. We cannot explain why we did not find MME in the other 4 ADOA patients in this study. It may be related to the disease severity; however, patient 2-II-1 showed comparable peripheral RNFL thickness and better BCVA compared to the other ADOA patients without MME. Some other factors may be needed for MME to develop.

In conclusion, our findings showed that genetically identified ADOA patients without any sign of inflammation can have MME in the INL of the retina. Our data indicate that the disease severity may be associated with the presence of MME in the INL as reported, because we found the clearest MME in the patients with poorest BCVA, although some other factors may be needed for MME to develop other than disease severity. Our findings indicate that there is a possibility that retrograde trans synaptic degeneration could cause severe damages in horizontal, bipolar, and amacrine cells in the INL after the optic nerve atrophy. Further studies are needed, and these findings will probably be helpful in clarifying the pathology of the degeneration of inner retinal cells by retrograde transsynaptic degeneration in patients with optic nerve atrophies and in developing new therapies.

## Figures and Tables

**Table 1:** Summary of the clinical data of patients with ADOA.

Patient ID	Sex	Age	BCVA^a^ (OD/OS)	Visual field	Disc appearance	Temporal RNFL^b^ thinning
1-II-1	F	35	0.08/0.07	Centrocecal scotoma	Temporal palor	Yes
2-II-1	M	39	0.3/0.4	Centrocecal scotoma	Temporal palor	Yes
3-III-1	F	43	0.2/0.4	Blind spot enlargement	Temporal palor	Yes
4-II-1	M	52	0.5/0.7	Blind spot enlargement	Temporal palor	Yes
4-III-1	F	20	0.7/0.6	Blind spot enlargement	Temporal palor	Yes
4-III-2	M	18	0.3/0.2	Centrocecal scotoma	Temporal palor	Yes

^a^Best corrected visual acuity (decimal).

^b^Retinal nerve fiber layer.

**Table 2:** Summary of the mutations of OPA1 gene.

Patient ID	Nucleotide change	Consequence^a^	Domain	Location	Reference
1-II-1	c.1771-1G>A	p.N591GfsX18 (splicing defect)	Dynamin central region	Boundary of intron 18- exon 19	This study
2-II-1	c.1899delT	p.I633MfsX12	Dynamin central region	Exon 20	This study
3-III-1	c.1096C>T	p.R366X	GTPase domain	Exon 11	Alexander et al. 2000 [10]
4-II-1	c.1102delT	p.R368GfsX4	GTPase domain	Exon 11	This study
4-III-1	Same as above				
4-III-2	Same as above				

^a^Reference sequence NM_015560.2.
